# The contribution of area-level walkability to geographic variation in physical activity: a spatial analysis of 95,837 participants from the 45 and Up Study living in Sydney, Australia

**DOI:** 10.1186/s12963-017-0149-x

**Published:** 2017-10-03

**Authors:** Darren J. Mayne, Geoffrey G. Morgan, Bin B. Jalaludin, Adrian E. Bauman

**Affiliations:** 1 Sydney School of Public Health, The University of Sydney, Camperdown, 2006 NSW Australia; 2 Public Health Unit, Illawarra Shoalhaven Local Health District, Warrawong, 2502 NSW Australia; 3 Graduate School of Medicine, University of Wollongong, Wollongong, 2500 NSW Australia; 4 Illawarra Health and Medical Research Institute, Wollongong, 2500 NSW Australia; 5 University Centre for Rural Health - North Coast, School of Public Health, The University of Sydney, Camperdown, 2006 NSW Australia; 6 Ingham Institute, University of New South Wales, Sydney, 2052 NSW Australia; 7 Epidemiology, Healthy People and Places Unit, Population Health, South Western Sydney Local Health District, Liverpool, 1871 NSW Australia

**Keywords:** Disease mapping, Geographic variation, Physical activity, Spatial model, Walkability

## Abstract

**Background:**

Individual-level studies support a positive relation between walkable built environments and participation in moderate-intensity walking. However, the utility of this evidence for population-level planning is less clear as it is derived at much finer spatial scales than those used for regional programming. The aims of this study were to: evaluate if individual-level relations between walkability and walking to improve health manifest at population-level spatial scales; assess the specificity of area-level walkability for walking relative to other moderate and vigorous physical activity (MVPA); describe geographic variation in walking and other MVPA; and quantify the contribution of walkability to this variation.

**Methods:**

Data on sufficient walking, sufficient MVPA, and high MVPA to improve health were analyzed for 95,837 Sydney respondents to the baseline survey of the 45 and Up Study between January 2006 and April 2010. We used conditional autoregressive models to create smoothed MVPA “disease maps” and assess relations between sufficient MVPA to improve health and area-level walkability adjusted for individual-level demographic, socioeconomic, and health factors, and area-level relative socioeconomic disadvantage.

**Results:**

Within-cohort prevalence of meeting recommendations for sufficient walking, sufficient MVPA, and high MVPA were 31.7 (95% CI 31.4–32.0), 69.4 (95% CI 69.1–69.7), and 56.1 (95% CI 55.8–56.4) percent. Prevalence of sufficient walking was increased by 1.20 (95% CrI 1.12–1.29) and 1.07 (95% CrI 1.01–1.13) for high and medium-high versus low walkability postal areas, and for sufficient MVPA by 1.05 (95% CrI 1.01–1.08) for high versus low walkability postal areas. Walkability was not related to high MVPA. Postal area walkability explained 65.8 and 47.4 percent of residual geographic variation in sufficient walking and sufficient MVPA not attributable to individual-level factors.

**Conclusions:**

Walkability is associated with area-level prevalence and geographic variation in sufficient walking and sufficient MVPA to improve health in Sydney, Australia. Our study supports the use of walkability indexes at multiple spatial scales for informing population-level action to increase physical activity and the utility of spatial analysis for walkability research and planning.

## Background

Promoting moderate-intensity walking is the cornerstone strategy of public health efforts to increase population levels of participation in moderate and vigorous physical activity (MVPA) [1–4]. Walking is low risk [5]; accessible to most people regardless of age, sex, socioeconomic status, or cultural background [2]; and confers health benefits independent of participating in more vigorous forms of physical activity [6]. Walking may be undertaken for recreation, leisure, and health; to move between destinations and origins; and to access services [7, 8]. These latter activities describe utilitarian walking or active transport, and have been a focus for built environment research over the last two decades [9]. “Walkability” is the term used to describe the capacity of built environments to facilitate walking for multiple purposes [8], especially active transport [10]. Walkable neighbourhoods facilitate active transport by reducing distances between origins and destinations, and maximizing the mix of proximal land uses for residential, commercial, educational, and recreational purposes [7, 10, 11].

Walkability is typically operationalized as an index of high-resolution built environment variables within a geographic information system. The most widely utilized and researched of these are the North American Neighborhood Quality of Life Study (NQLS) [12] and South Australian Physical Activity in Localities and Community Environments (PLACE) [8] indexes. Developed in parallel for comparing between North American and Australian populations [8], these metrics comprise four environmental variables: residential density, street network connectivity, land use mix, and retail floor area ratio, which are combined into a total score by summing over either decile ranks [8] or standard (Z) scores [12]. The total score is then divided into sample-specific quartiles representing the relative variation in walkability between units of analysis [8, 12]. The indexes have been adapted and validated for use in other cities and countries (e.g. [8, 9, 13–19]), and underpin a large international evidence base demonstrating consistent associations between environmental walkability and levels of moderate-intensity walking that benefits health [17].

Creating local opportunities for transport-related walking through strategic land use and infrastructure developments is a key strategy of many regional development plans [9, 17] such as the Sydney Metropolitan Plan [20], and may contribute to population levels of total daily physical activity [3, 4, 7]. Environmental and policy interventions such as these generally have much smaller effect sizes than those targeting individuals [21] but can have larger population-level impacts because exposure to the built environment is ubiquitous and changes more persistent than interventions with individuals [7, 22, 23]. This has prompted some to recommend using walkability indexes to inform urban design, transportation, and health policy; target infrastructure investments; and evaluate environmental interventions to increase population-levels of physical activity [8, 9, 17, 24–27].

Needs assessment, planning, intervention, and evaluation activities to address the health of populations typically occur at larger regional levels [28], which Saelens and Handy have termed macro-level environments [10]. These are distinct from micro-level environments specific to individuals and meso-level environments that are shared by groups of individuals, such as residents within a neighborhood [10]. Greater use of objective walkability indexes has demonstrably progressed our understanding of environment-behavior relations through a focusing of research and inference at increasingly finer spatial resolutions (see reviews [10, 11, 29–31]). However, this has contributed to an evidence base derived at geographic scales substantially smaller than those used for population health policy, planning, and intervention; assumes individual-level environment-behavior relations scale to populations; and raised concerns about the utility of micro-level evidence for macro-level health programming [32].

The extent to which micro-level correlates of physical activity manifest at macro-levels is poorly understood [33], under-researched, and limited to journey to work data among employed populations. For example, Frank et al. have reported that the prevalence of employed persons walking to work at the 2000 United States Census was 4-7% higher in the most compared to least walkable block groups of King County and Baltimore after stratifying on household income [12]. We too have observed higher prevalence of income-stratified self-reported walking to work in the 2006 Australian Census for Sydney residents in the most (7.9-11.0%) versus least (2.1-3.0%) walkable Census Collection Districts and adjusted odds of walking to work 2.8-3.3 times higher for the top versus bottom walkability quartiles [17]. Similarly, Kelly et al. have recently reported that the odds of ≥5% of employed block group residents reporting walking to work in the 2004–2009 American Community Survey were 1.6-5.5 times higher for the most compared to least walkable neighborhoods in St. Louis City and County after adjusting for area-level socioeconomic deprivation [34]. These small number of macro-level built environment studies are consistent with a positive association between population-prevalence of utilitarian walking and area-level walkability. However, the behavioral specificity of outcome measures for walking to work among employed persons in these studies limit their generalizability to the broader population and other domains of walking.

Walkability research has also largely ignored geographic variation in behaviors and outcomes, which is essential for framing public policy and action [35]. Spatial variation in outcomes and behavior beyond that explained by demographic, social, and economic factors may indicate additional, unobserved, and geographically varying influences on health and health-enhancing infrastructure [36]. Spatial analysis is also a potent tool for identifying environmental inequalities [37] and may assist in targeting infrastructure upgrades and developments to mitigate environment-related health risks, and promote equitable access to health-enhancing built environments [38, 39]. For example, Huang and colleagues have identified clusters of high active transportation (walking or riding) among residents of Los Angeles and San Diego Counties living in Census block groups with higher population, employment, street, block, and intersection densities; shorter block lengths; and the presence of a bus route [38]. Tamura et al. have also observed significant clustering of prevalence of meeting physical activity guidelines in California, Massachusetts, and Pennsylvania respondents to the 2004 Nurses’ Health Study [39]; however, they found inconsistent evidence for macro-level differences between cluster and non-cluster neighborhoods [39].

Given the limited evidence on macro-level relationships between walking and walkability, and the potential for geographic analysis to inform this research, the aims of the present study were to: (1) evaluate if area-level walkability was associated with population-levels of moderate-intensity walking; (2) assess the specificity of area-level walkability for walking compared to other MVPA; (3) describe geographic variation in walking and other MVPA; and (4) quantify the contribution of area-level walkability to this variation using a population-based cohort living in Sydney, Australia. We hypothesized that: (1) area-level walkability would be associated with population-levels of sufficient walking to improve health but not other MVPA; (2) sufficient walking and other MVPA to improve health would vary geographically in the study area; and (3) area walkability would contribute to this variation but only for sufficient walking to improve health.

## Methods

### Study design and area

We used a cross-sectional, ecological design to investigate geographic variation in physical activity behavior and its relationship to walkability in the Sydney Statistical Division of NSW, Australia [40]. Sydney covers a land area of 12142 km^2^ and had a population of 4.1 million persons living in 1.6 million dwellings at the 2006 Census [41]. Analysis was undertaken at the Census postal area level, which was the smallest spatial unit at which geographically identified 45 and Up Study data were available from the data custodian. Sydney comprised 260 conterminous postal areas at the 2006 Census [42] with a median land area of 7.6 km^2^, 5304 residential dwellings, and 13090 residents [41].

### Participants

Participants were selected from members of the The Sax Institute’s 45 and Up Study, a population-based cohort established between January 2006 and December 2010 to investigate healthy aging among persons 45 years and older living in NSW, Australia [43]. Prospective participants were randomly sampled from the Medicare Australia enrollment database and invited to return a completed consent form and baseline questionnaire via mail [43]. People aged ≥80 years were oversampled by a factor of two; rural and remote populations were also oversampled but these subgroups are not resident within the Sydney area [43]. The 45 and Up Study includes approximately 10% of the NSW population and had a response rate of 18% [43]. This is similar to other international population cohorts that seek consent for data linkage (e.g., [44, 45]), and consistent with the global trend of reducing participation rates in epidemiological studies [46]. Individual-level data were provided by The Sax Institute with 2006 Census postal areas identifiers for all 266848 persons recruited to the study between January 2006 and April 2010 [47]. We limited our analysis to the 115153 persons from this release that were geocoded to the Sydney Statistical Division to correspond with the spatial extent of our study factor.

### Data

Individual-level data comprised self-reported responses to the baseline questionnaire of the 45 and Up Study [43], and were used to derive respondent-level physical activity outcome variables and covariates. Postal area-level data included the Sydney Walkability Index (SWI) [17] and the 2006 Index of Relative Socioeconomic Disadvantage (IRSD) [48], which were included as study and covariate factors, respectively.

### Outcome variables

We defined three physical activity outcomes: sufficient moderate-intensity walking to improve health, sufficient MVPA to improve health, and high MVPA. Each outcome was derived from self-reported responses to Active Australia Survey [49] questions included in the baseline survey. Participants were asked to report separately the number of times in the last week they had: (i) walked continuously for at least 10 minutes for recreation or exercise or to get to or from places; (ii) participated in vigorous physical activity that made them breathe harder or puff or pant; (iii) participated in other moderate physical activities. Participants also reported the total time they spent doing each of these activities.

#### Sufficient total moderate and vigorous physical activity to improve health

Moderate and vigorous physical activity was calculated as the sum of total minutes engaged in walking and other moderate-intensity activities plus two times the total number of minutes engaged in vigorous physical activities. Respondents were classified as sufficiently active if they accumulated ≥150 min of MVPA over ≥5 sessions of at least 10 minutes duration. A double weighting is given to vigorous activity to reflect its greater intensity, and a threshold of five sessions operationalizes the Australian physical activity guidelines recommendation that adults be active on most days of the week, and assumes sessions are most likely to occur on separate days [50].

#### Sufficient walking to improve health

Sufficient walking to improve health used the same frequency and duration criteria as sufficient MVPA but only used responses on walking in calculations. This outcome measure identified respondents that met Australian physical activity guidelines from walking alone. We included this outcome on the basis that walkability indexes have higher specificity for walking behavior—especially for utilitarian purposes—than for MVPA [8, 12, 17].

#### High moderate and vigorous physical activity

We categorized respondents as highly physically active if they reported participating in ≥300 minutes and ≥5 sessions of MVPA over the previous week. The purpose of this outcome was to identify respondents at or above the upper Australian guideline limit of 300 minutes of MVPA per week [51]. This represents a level of MVPA at which even more health gains are accrued [52] and potentially a minimum level of MVPA required for certain health outcomes such as prevention of weight gain and some cancers [53].

### Study variable

The study variable of interest was postal area walkability measured using the SWI [17]. The SWI is an abridged version of the NQLS and PLACE indexes [17]. It has predictive validity for utilitarian walking, and is a cohesive and internally consistent measure of walkability in Sydney, Australia [17]. The index is derived within a geographical information system and operationalizes three measures of the built environment associated with walking:

1. Residential dwelling density—number of residential dwellings per hectare of residential land use
2. Intersection density—number of intersections with three or more road junctures per square kilometre of total land area
3. Land use mix—entropy of five land use classes (residential, commercial, industrial, recreational, and other uses) adjusted for differences in the size of spatial units [54].

Variable values are divided into deciles, scored from 1 (lowest) to 10 (highest), summed to give a total score out of 30, which is then divided into quartiles corresponding to low, low-medium, medium-high, and high walkability [17]. We have previously demonstrated that abridged indexes excluding retail floor space ratio data, which are difficult to source [8, 12] and frequently unavailable [55], have comparable measurement properties to four-variable NQLS and PLACE indexes [17].

We calculated SWI variables within the spatial extents of Sydney postal areas, which have a median land area of 7.6 km^2^ that approximately corresponds to a radial buffer of 1550 meters. This is within the range of “high resolution” buffers typically used for individual-level analyses, [56, 57] and for which consistent environment-behavior associations have been reported across adult life stages, including older adulthood [56].

### Covariates

We included individual and area-level correlates and determinants of physical activity from the research and 45 and Up Study literatures (e.g. [58–64]). Individual-level covariates included sex; age at baseline interview; language spoken at home; education level; relationship status; employment status; health insurance type; smoking status; body mass category; number of chronic conditions ever diagnosed and number of chronic conditions treated in the last month; and physical function limitation and role limitation due to emotional problems sub-scales from the RAND 36-Item Health Survey (Version 1.0) [65].

Area-level socioeconomic disadvantage was measured for postal areas using the 2006 IRSD [48]. The IRSD is a general measure of disadvantage derived from Census variables indicative of low socioeconomic well being such as percent of population ≥15 years with no post school qualification; percent of population unemployed; percent of employed persons classified as laborers; percent of private dwellings with no motor car; and percent of people who do not speak English. We divided the postal area distribution of IRSD scores into quintiles indicating high through to low relative socioeconomic disadvantage.

### Statistical analysis

We used a two-stage strategy to model within-cohort prevalence. In the first stage predicted probabilities ($\hat {Y}_{ij}$) of achieving sufficient walking, sufficient MVPA and high MVPA to improve health were calculated for each respondent using logistic regression models conditioned on individual-level covariates. These probabilities were summed within the *j* postal areas to obtain the predicted number of outcomes for each unit adjusted for its underlying respondent structure [66–68].

In the second stage postal area prevalence ratios (PR) for physical activity outcomes were estimated using Bayesian Besag, York, and Mollié conditional autoregressive models with Poisson likelihoods [69]. This model is commonly used in epidemiology for small-area disease mapping estimation [70, 71] and decomposes area-specific random effects into a local, spatially structured variance component (*s*_*j*_) and a global, spatially unstructured (heterogeneity) variance component (*u*_*j*_) [70, 72]:

1 $$ log(\theta_{j}) = \alpha + x_{j}\beta + s_{j} + u_{j} + log(e_{j})  $$

where *θ*_*j*_ is the relative risk for the *j* ^*th*^ area; *α* is the overall relative risk across the study region; *x*_*j*_ and *β* are optional vectors of ecological explanatory variables and parameter estimates, respectively; and *e*_*j*_ is an offset representing the expected number of cases in the *j* ^*th*^ area, which we derive using either the overall prevalence (*e*_*j*_=*p*×*n*_*j*_) or sum of predicted probabilities from stage one ($e_{j} = \sum \hat {Y}_{ij}$).

The heterogeneity component (*u*_*j*_) was given a normal prior with mean 0 and precision $\tau _{u}^{2}$ [70]. The local smoothing component (*s*_*i*_) was given an intrinsic conditional autoregressive prior [70] with mean $\bar {s}_{j}$ and precision ${tau}_{j}^{2}$ conditioned on the mean risk in the *k* surrounding postal areas with intersecting boundaries. Variability of *u*_*j*_ and *s*_*j*_ were controlled by hyper-parameters $\tau _{u}^{2}$ and $\tau _{s}^{2}$ [70], which were given Gamma hyper-priors of *γ*(0.5,0.0005) [73].

Six models were fit for each outcome. Model 1 (M1) was an unadjusted disease mapping model with expected cases proportional to the overall prevalence (*p*×*n*_*j*_). Model 2 (M2) was also a disease mapping model but with individually-adjusted expected cases from stage 1. Models 3-6 were ecological regressions. Model 3 (M3) added SWI to M2; Model 4 (M4) added IRSD to M2; Model 5 (M5) added IRSD to M3; and Model 6 (M6) added effect modification of SWI by IRSD to M5.

Medians and 95% credible intervals (CrI) for each model parameter were summarized from the posterior distribution obtained from two Monte Carlo Markov Chains with over-dispersed starting values. Each chain ran for 2.5 million iterations with every 250^*th*^ sample retained to reduce autocorrelation and improve convergence. The first half of each chain was discarded as burn-in leaving 10000 samples in total for inference. Model convergence was assessed using trace and autocorrelation plots, and Gelman-Rubin diagnostics [74].

The *Deviance Information Criterion* (DIC) was used to choose between spatial models [70] and evaluate the importance of area-level variables [75]. We considered models within 1–2 DIC units of the best model (i.e., lowest DIC) as deserving consideration, 3–7 as having less support, and >7 no support [76]. An increase in DIC between nested models was interpreted as support for selecting the variable omitted from the reduced model [75]. We visualized the exponentiated sum of spatial and non-spatial variance components using choropleth maps to identify variation in excess of that attributable to fixed-effect factors. We also calculated the spatial fraction $\left ({\rho = \sigma ^{2}_{s}/}\left [{\sigma ^{2}_{s} + \sigma ^{2}_{u}}\right ]\right)$ from the marginal variation of random effects to determine the proportion of residual variation due to spatially-structured factors [77, 78].

#### Weighting

Weighting of the 45 and Up Study sample is not required to estimate externally valid relative effect measures when non-spatial analyses condition on the variables used to construct post-stratification weights [79]. However, whether weighting is necessary for valid geographical analysis of the cohort is unclear. Unweighted spatial analyses are simpler to implement within a Bayesian disease mapping framework [80, 81] but geographically structured (non) response rates can bias inference [82]. In the context of our study, this potential for bias arises through the estimation of postal area prevalence ratios from sample counts and expectations. We evaluated the need to adjust our sample for response rates by comparing postal area prevalence ratios derived using unweighted and weighted sample data for each physical activity outcome. Post-stratification survey weights were calculated to benchmark the study sample to the Sydney Statistical Division population from the 2006 Census [83], with post-strata formed by 2006 Census postal areas (n=260), sex (male and female), and five-year age groups (45–84 and ≥85 years). We evaluated the performance of unweighted data using scatter plots and Pearson correlation coefficients to visualize relationships and strength of associations with weighted postal area prevalence ratios calculated using both unadjusted and adjusted expected cases.

All data analysis and mapping was undertaken in R 3.2.2 using R2WinBUGS 2.1-21 and sp 1.2-1. Correlation coefficients, t-tests, and general linear models were evaluated at the 5% alpha level and conditional autoregressive models using DIC and 95% credible intervals summarized from posterior distributions.

## Results

Complete data were available for 95837 of 115153 (83.2%) cohort members living in 255 of 260 (98.1%) postal areas in Sydney. Respondent counts within postal areas ranged from 0–3481 with a median and interquartile range of 271 and 152-466. Sample characteristics for people included in our study are reported in Table 2. Gender and employment status were comparable to 2006 Census estimates for the study area [83]; however, similar to the full cohort profile [47], our sample was younger, more highly educated, less likely to speak a language other than English at home, and more likely to be living with a partner than the study population.

### Walkability

Median walkability scores for low, low-medium, medium-high, and high walkability quartiles were 5, 13, 19, 27, respectively. Table 1 reports the median, minimum and maximum values for environmental variables by walkability quartiles. Variable values increased monotonically but non-linearly between successively increasing walkability quartiles. The ratios of environmental median values for high compared to low walkability quartiles were approximately two times higher than for medium-high compared to low quartiles, which were approximately two times higher than for low-medium compared to low quartiles. Interquartile ranges for residential density (0.74–7.25, 9.63–15.62, 16.95–22.31, and 36.60–66.91), intersection density (1.94–10.34, 34.28–59.01, 66.87–89.86, and 121.50–203.00), and land use mix (0.002–0.014, 0.020–0.048, 0.047–0.081, and 0.088–0.218) did not overlap when stratified by low, low-medium, medium-high, and high walkability quartiles, and indicates that each variable is contributing to the segmentation of postal area walkability.

**Table 1:** Median, low, and high values for Sydney Walkability Index environmental variables by walkability quartiles

Walkability	Residential density per ha				Intersection density per km^2^				Land use mix entropy		
	Median	Low	High		Median	Low	High		Median	Low	High
Low	2.28	0.11	18.06		3.37	0.08	37.18		0.005	0.000	0.067
Low-medium	13.35	0.00	28.51		46.14	1.53	102.20		0.033	0.001	0.218
Medium-high	19.82	11.96	55.32		79.53	6.44	117.80		0.056	0.030	0.400
High	46.02	22.02	219.70		162.50	80.87	695.10		0.134	0.045	0.631

### Prevalence of physical activity outcomes

Prevalence of sufficient walking, sufficient MVPA, and high MVPA to improve health were 31.7 (95% CI 31.4-32.0), 69.4 (95% CI 69.1-69.7), and 56.1 (95% CI 55.8-56.4) percent, respectively. Frequencies, relative frequencies, and prevalence of physical activity outcomes for area-level factors are reported at the top of Table 2. Sufficient walking to improve health displayed the strongest prevalence gradient for area-level walkability followed by sufficient MVPA; the gradient for high MVPA was small and inconsistent. Prevalence of all outcomes increased with increasing area-level socioeconomic disadvantage but the gradient was weakest for sufficient walking to improve health.

**Table 2:** Sample characteristics and prevalence estimates for physical activity outcomes

Variable	Characteristics			Prevalence					
				Sufficient walking		Sufficient MVPA		High MVPA	
	N	%		n	%	n	%	n	%
POSTAL AREA LEVEL									
*Walkability*									
Low	26435	27.6		7582	28.7	18079	68.4	14763	55.8
Low-medium	32696	34.1		9854	30.1	22225	68.0	17961	54.9
Medium-high	20299	21.2		6565	32.3	14119	69.6	11388	56.1
High	16407	17.1		6353	38.7	12087	73.7	9668	58.9
*Socioeconomic disadvantage*									
Q1 - Most	18263	19.1		5334	29.2	11690	64.0	9300	50.9
Q2	20349	21.2		6105	30.0	13610	66.9	10882	53.5
Q3 - Middling	15575	16.3		5025	32.3	10736	68.9	8674	55.7
Q4	20723	21.6		6999	33.8	15203	73.4	12510	60.4
Q5 - Least	20927	21.8		6891	32.9	15271	73.0	12414	59.3
INDIVIDUAL LEVEL									
*Sex*									
Male	46099	48.1		14748	32.0	31432	68.2	25039	54.3
Female	49738	51.9		15606	31.4	35078	70.5	28741	57.8
*Age*									
45-49	13605	14.2		3865	28.4	9462	69.5	7590	55.8
50-54	16843	17.6		5169	30.7	11996	71.2	9576	56.9
55-59	17008	17.7		5645	33.2	12108	71.2	9777	57.5
60-64	14114	14.7		4922	34.9	10307	73.0	8497	60.2
65-69	10703	11.2		3747	35.0	7772	72.6	6468	60.4
70-74	7387	7.7		2541	34.4	5250	71.1	4306	58.3
75-79	5519	5.8		1724	31.2	3662	66.4	2941	53.3
80-84	7464	7.8		2057	27.6	4403	59.0	3460	46.4
≥85	3194	3.3		684	21.4	1550	48.5	1165	36.5
*Language spoken at home*									
English	81196	84.7		25937	31.9	57486	70.8	46737	57.6
Other	14641	15.3		4417	30.2	9024	61.6	7043	48.1
*Education level*									
Less than secondary school	8057	8.4		2184	27.1	4508	56.0	3641	45.2
Secondary school graduation	28177	29.4		8566	30.4	18790	66.7	15240	54.1
Trade, certificate, or diploma	30119	31.4		9534	31.7	21263	70.6	17400	57.8
University degree	29484	30.8		10070	34.2	21949	74.4	17499	59.4
*Relationship status*									
Partner	71083	74.2		22315	31.4	50009	70.4	40528	57.0
No partner	24754	25.8		8039	32.5	16501	66.7	13252	53.5
*Employment status*									
Full-time work	33116	34.6		9958	30.1	22750	68.7	17796	53.7
Part-time work	13509	14.1		4287	31.7	9925	73.5	8130	60.2
Other work	1417	1.5		492	34.7	1044	73.7	892	62.9
Not working	47795	49.9		15617	32.7	32791	68.6	26962	56.4
*Health insurance type*									
Private with extras	55802	58.2		17921	32.1	40165	72.0	32568	58.4
Private without extras	13597	14.2		4347	32.0	9574	70.4	7803	57.4
Government health care card	12977	13.5		3887	30.0	7928	61.1	6320	48.7
None	13461	14.0		4199	31.2	8843	65.7	7089	52.7
*Smoking status*									
Never smoked	56362	58.8		17651	31.3	39255	69.6	31582	56.0
Past smoker	32897	34.3		10773	32.7	23196	70.5	18923	57.5
Current smoker	6578	6.9		1930	29.3	4059	61.7	3275	49.8
*Body mass category*									
Underweight	1360	1.4		484	35.6	877	64.5	717	52.7
Normal weight	37712	39.4		13179	34.9	27771	73.6	22833	60.5
Overweight	37271	38.9		11902	31.9	26169	70.2	21165	56.8
Obese	19494	20.3		4789	24.6	11693	60.0	9065	46.5
*Diagnosed chronic conditions*									
0	32167	33.6		10442	32.5	23287	72.4	19019	59.1
1	38557	40.2		12403	32.2	26887	69.7	21749	56.4
2	19082	19.9		5873	30.8	12679	66.4	10114	53.0
3 or more	6031	6.3		1636	27.1	3657	60.6	2898	48.1
*Treated chronic conditions*									
0	42523	44.4		13610	32.0	30498	71.7	24891	58.5
1	31399	32.8		10328	32.9	22098	70.4	17788	56.7
2	15478	16.2		4709	30.4	10110	65.3	8066	52.1
3 or more	6437	6.7		1707	26.5	3804	59.1	3035	47.1
*Functional limitation*									
None	33079	34.5		11648	35.2	25449	76.9	21427	64.8
Minor	25661	26.8		9250	36.0	19221	74.9	15500	60.4
Moderate	21192	22.1		6488	30.6	14159	66.8	11038	52.1
Severe	15905	16.6		2968	18.7	7681	48.3	5815	36.6
*Emotional problems*									
None	67240	70.2		22286	33.1	47682	70.9	38888	57.8
Minor	13394	14.0		4005	29.9	9365	69.9	7495	56.0
Moderate	7638	8.0		2144	28.1	5018	65.7	3941	51.6
Severe	7565	7.9		1919	25.4	4445	58.8	3456	45.7

MVPA Moderate and vigorous physical activity, N Stratum total, n Stratum outcome frequency, % Stratum outcome percent

### Individual-level factors

Sample characteristics and prevalence of study outcomes for individual-level factors are reported in Table 2 and full-model odds ratio estimates in Table 3. Physical activity outcomes were strongly associated with individual-level demographic, social, economic, and health status and behavior factors. For all outcomes, prevalence of sufficient MVPA increased with increasing education level; decreased with increasing numbers of diagnosed and treated chronic health conditions, functional limitation and emotional problems; and were higher for females, people who spoke English at home, or were non- or ex-smokers. These gradients and differences were less pronounced for sufficient walking to improve health than for either sufficient MVPA or high MVPA. This was also observed for age, which displayed inverted U-shape associations with prevalence of MVPA outcomes. Prevalence of sufficient walking to improve health decreased with increasing body mass and was especially low in obese persons; body mass gradients were less consistent for sufficient MVPA and high MVPA. Health insurance type was unrelated to prevalence of sufficient walking to benefit health but strongly related to both sufficient MVPA and high MVPA prevalence. The areas under the curve for fully adjusted individual-level logistic regression models were 61.0%, 66.6%, and 64.9% for sufficient walking, sufficient MVPA, and high MVPA, respectively.

**Table 3:** Full-model odds ratio estimates for individual-level adjustment variables

	Sufficient walking			Sufficient MVPA			High MVPA	
	OR	95% CI		OR	95% CI		OR	95% CI
*Sex*	*p*=0.0407			*p*<0.0001			*p*<0.0001	
Male	1.00			1.00			1.00	
Female	0.99	(0.96-1.02)		1.20	(1.16-1.24)		1.21	(1.17-1.25)
*Age*	*p*<0.0001			*p*<0.0001			*p*<0.0001	
45-49	1.00			1.00			1.00	
50-54	1.13	(1.08-1.19)		1.13	(1.08-1.19)		1.09	(1.04-1.14)
55-59	1.25	(1.19-1.32)		1.13	(1.08-1.20)		1.11	(1.06-1.16)
60-64	1.27	(1.20-1.34)		1.16	(1.10-1.23)		1.15	(1.09-1.21)
65-69	1.20	(1.13-1.28)		1.08	(1.01-1.16)		1.09	(1.03-1.16)
70-74	1.18	(1.10-1.27)		1.06	(0.98-1.14)		1.04	(0.97-1.12)
75-79	1.06	(0.98-1.15)		0.90	(0.83-0.98)		0.90	(0.84-0.98)
80-84	0.93	(0.86-1.01)		0.71	(0.66-0.76)		0.74	(0.69-0.79)
≥85	0.73	(0.66-0.81)		0.53	(0.48-0.58)		0.55	(0.50-0.61)
*Language spoken at home*	*p*<0.0001			*p*<0.0001			*p*<0.0001	
English	1.00			1.00			1.00	
Other	0.70	(0.93-1.00)		0.70	(0.67-0.73)		0.72	(0.69-0.75)
*Education level*	*p*<0.0001			*p*<0.0001			*p*<0.0001	
University degree	1.00			1.00			1.00	
Trade, certificate, or diploma	0.92	(0.89-0.95)		0.90	(0.87-0.94)		1.01	(0.98-1.04)
Less than secondary school	0.88	(0.85-0.91)		0.76	(0.73-0.79)		0.87	(0.84-0.90)
Secondary school graduation	0.81	(0.77-0.86)		0.59	(0.56-0.62)		0.72	(0.68-0.76)
*Relationship status*	*p*<0.0001			*p*<0.0001			*p*<0.0001	
Partner	1.00			1.00			1.00	
No partner	1.18	(1.14-1.22)		1.04	(1.00-1.07)		1.02	(0.99-1.05)
*Employment status*	*p*<0.0001			*p*<0.0001			*p*<0.0001	
Full-time work	1.00			1.00			1.00	
Part-time work	1.10	(1.05-1.15)		1.25	(1.19-1.32)		1.27	(1.21-1.33)
Other work	1.46	(1.30-1.64)		1.81	(1.59-2.06)		1.93	(1.72-2.17)
Not working	1.36	(1.30-1.42)		1.67	(1.59-1.75)		1.73	(1.66-1.81)
*Health insurance type*	*p*=0.0564			*p*<0.0001			*p*<0.0001	
Private with extras	1.00			1.00			1.00	
Private without extras	1.00	(0.96-1.04)		0.97	(0.93-1.01)		0.99	(0.95-1.03)
Government health care card	1.10	(1.05-1.15)		0.96	(0.92-1.01)		0.95	(0.91-0.99)
None	1.05	(1.01-1.10)		0.91	(0.87-0.95)		0.92	(0.89-0.96)
*Smoking status*	*p*<0.0001			*p*<0.0001			*p*<0.0001	
Never smoked	1.00			1.00			1.00	
Past smoker	1.11	(1.07-1.14)		1.15	(1.12-1.19)		1.16	(1.13-1.20)
Current smoker	0.97	(0.92-1.03)		0.82	(0.78-0.87)		0.90	(0.86-0.96)
*Body mass category*	*p*<0.0001			*p*<0.0001			*p*<0.0001	
Underweight	1.18	(1.05-1.33)		0.81	(0.72-0.91)		0.87	(0.77-0.97)
Normal weight	1.00			1.00			1.00	
Overweight	0.87	(0.84-0.90)		0.86	(0.83-0.89)		0.89	(0.86-0.91)
Obese	0.66	(0.63-0.69)		0.62	(0.60-0.65)		0.66	(0.64-0.69)
*Diagnosed chronic conditions*	*p*<0.0001			*p*<0.0001			*p*<0.0001	
0	1.00			1.00			1.00	
1	1.04	(1.01-1.08)		1.00	(0.96-1.03)		1.00	(0.97-1.04)
2	1.07	(1.02-1.12)		1.00	(0.96-1.04)		1.00	(0.96-1.04)
3 or more	1.01	(0.94-1.08)		0.91	(0.85-0.97)		0.94	(0.88-1.00)
*Treated chronic conditions*	*p*<0.0001			*p*<0.0001			*p*<0.0001	
0	1.00			1.00			1.00	
1	1.06	(1.03-1.10)		1.05	(1.01-1.09)		1.00	(0.97-1.04)
2	1.04	(0.99-1.09)		0.97	(0.93-1.02)		0.95	(0.91-0.99)
3 or more	0.99	(0.93-1.06)		0.91	(0.86-0.97)		0.92	(0.87-0.98)
*Functional limitation*	*p*<0.0001			*p*<0.0001			*p*<0.0001	
None	1.00			1.00			1.00	
Minor	1.01	(0.97-1.04)		0.88	(0.84-0.91)		0.80	(0.77-0.83)
Moderate	0.79	(0.76-0.82)		0.62	(0.59-0.65)		0.58	(0.56-0.60)
Severe	0.42	(0.40-0.44)		0.32	(0.30-0.34)		0.33	(0.32-0.35)
*Emotional problems*	*p*<0.0001			*p*<0.0001			*p*<0.0001	
None	1.00			1.00			1.00	
Minor	0.90	(0.86-0.94)		1.04	(0.99-1.08)		1.01	(0.97-1.05)
Moderate	0.88	(0.83-0.93)		0.95	(0.90-1.00)		0.93	(0.88-0.98)
Severe	0.84	(0.80-0.89)		0.84	(0.80-0.89)		0.84	(0.80-0.89)

MVPA Moderate and vigorous physical activity, OR Odds ratio, CI Confidence interval

### Weighting

**Fig. 1:**
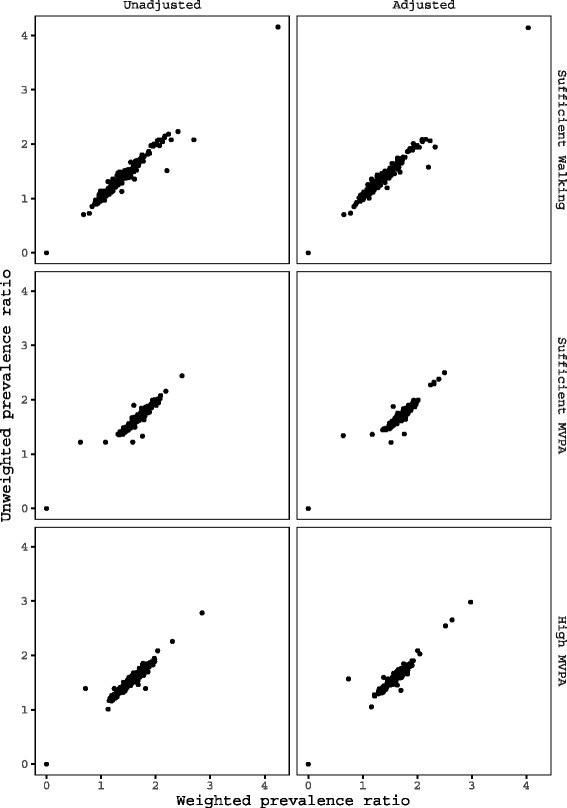
Comparison of unweighted and post-strata weighted postal area prevalence ratios for physical activity outcomes in Sydney Statistical Division. Post-strata were formed by postal areas (N=260), sex (male and female), and five-year age groups (45–84 and ≥85 years). Left panel plots show relationships for prevalence ratios with no adjustment for individual-level factors. Right panel plots show relationships for prevalence ratios adjusted for individual-level demographic, economic, and health factors using logistic regressions

Figure 1 summarizes relationships between unweighted and weighted physical activity prevalence ratios for the 255 postal areas for which survey data were available. Left panel plots show prevalence ratios derived using unadjusted expected values, and right panel plots show prevalence ratios derived using expected values adjusted for individual-level factors. Strong linear relationships were observed between unweighted and weighted prevalence ratios for all physical activity outcomes, regardless of the method used to derive expected cases. Correlation coefficients for sufficient walking, sufficient MVPA, and high MVPA were 0.98, 0.96, and 0.97 for unadjusted, and 0.98, 0.94, and 0.96 for adjusted prevalence ratios, respectively. Based on these strong relations, we determined that weighting was not necessary for spatial analyses, and fit disease mapping models to unweighted sample data.

### Spatial analysis

Tables 4, 5 and 6 summarize conditional autoregressive models 1–5 for sufficient walking, sufficient MVPA, and high MVPA to improve health, respectively. Mean baseline models (M1) indicated very high levels of clustering with ≥97% of residual variation due to unobserved, spatially structured factors. Differences in effective parameters (pD) and DIC values indicated that the addition of expected cases adjusted for respondent-level variables (M2) simplified models and substantially improved fits over M1 for sufficient walking (*Δ*_*DIC*_=-16.3), sufficient MVPA (*Δ*_*DIC*_=-76.9), and high MVPA (*Δ*_*DIC*_=-76.7). These were the best fitting models for sufficient MVPA and high MVPA, and reduced spatial variation by 84.2% and 82.2%, respectively. The best fitting model for sufficient walking was M5, which included SWI and IRSD, and reduced the DIC and spatial variance by 20.8% and 75.6% over M1, respectively.

**Table 4:** Conditional autoregressive model summaries for sufficient walking to improve health

	Model 1	Model 2	Model 3	Model 4	Model 5
Individual-level adjustment	No	Yes	Yes	Yes	Yes
Parameter estimates (PR, 95% CrI)					
Constant	1.00 (0.99-1.02)	1.01 (0.99-1.02)	0.94 (0.90-0.98)	1.03 (1.00-1.08)	0.97 (0.91-1.03)
Walkability					
Low	–	–	1.00	–	1.00
Low-medium	–	–	1.03 (0.99-1.08)	–	1.03 (0.98-1.07)
Medium-high	–	–	1.07 (1.01-1.13)	–	1.05 (0.99-1.11)
High	–	–	1.20 (1.12-1.29)	–	1.18 (1.09-1.27)
Socioeconomic disadvantage					
High	–	–	–	1.00	1.00
High-medium	–	–	–	0.98 (0.93-1.03)	0.98 (0.94-1.03)
Medium	–	–	–	0.99 (0.94-1.04)	1.00 (0.95-1.05)
Medium-low	–	–	–	0.97 (0.91-1.02)	0.97 (0.92-1.03)
Low	–	–	–	0.92 (0.86-0.98)	0.94 (0.89-1.00)
Model diagnostics					
pD	92.37	75.41	62.05	76.81	65.25
DIC	1875.16	1858.87	1855.11	1857.33	1854.39
Fit (1=best, 5=poorest)	5	4	2	3	1
Spatial fraction	0.98	0.97	0.90	0.97	0.93

PR prevalence ratio, CrI credible interval, pD effective parameters, DIC Deviance Information Criterion

Model 1 null model with expected cases proportional to the overall prevalence

Model 2 null model with expected cases adjusted for individual-level factors

Model 3 Model 2 + Sydney Walkability Index

Model 4 Model 2 + Index of Relative Socioeconomic Disadvantage

Model 5 Model 3 + Index of Relative Socioeconomic Disadvantage

**Table 5:** Conditional autoregressive model summaries for sufficient MVPA to improve health

	Model 1	Model 2	Model 3	Model 4	Model 5
Individual-level adjustment	No	Yes	Yes	Yes	Yes
Parameter estimates (PR, 95% CrI)					
Constant	0.99 (0.99-1.00)	1.00 (0.99-1.01)	0.99 (0.97-1.01)	1.00 (0.97-1.02)	0.98 (0.96-1.01)
Walkability					
Low	–	–	1.00	–	1.00
Low-medium	–	–	1.00 (0.98-1.02)	–	1.00 (0.97-1.02)
Medium-high	–	–	1.01 (0.98-1.04)	–	1.01 (0.98-1.04)
High	–	–	1.05 (1.01-1.08)	–	1.04 (1.01-1.08)
Socioeconomic disadvantage					
High	–	–	–	1.00	1.00
High-medium	–	–	–	1.00 (0.97-1.03)	1.00 (0.97-1.02)
Medium	–	–	–	1.00 (0.97-1.03)	1.00 (0.97-1.03)
Medium-low	–	–	–	1.02 (0.99-1.06)	1.02 (0.99-1.05)
Low	–	–	–	1.00 (0.97-1.03)	1.00 (0.97-1.03)
Model diagnostics					
pD	67.54	30.68	28.77	32.31	30.16
DIC	1983.08	1906.15	1906.62	1909.87	1909.89
Fit (1=best, 5=poorest)	5	1	2	3	4
Spatial fraction	0.97	0.85	0.75	0.83	0.69

PR prevalence ratio, CrI credible interval, pD effective parameters, DIC Deviance Information Criterion

Model 1 null model with expected cases proportional to the overall prevalence

Model 2 null model with expected cases adjusted for individual-level factors

Model 3 Model 2 + Sydney Walkability Index

Model 4 Model 2 + Index of Relative Socioeconomic Disadvantage

Model 5 Model 3 + Index of Relative Socioeconomic Disadvantage

**Table 6:** Conditional autoregressive model summaries for high MVPA

	Model 1	Model 2	Model 3	Model 4	Model 5
Individual-level adjustment	No	Yes	Yes	Yes	Yes
Parameter estimates (PR, 95% CrI)					
Constant	0.99 (0.98-1.00)	1.00 (0.99-1.01)	0.99 (0.97-1.02)	0.99 (0.96-1.01)	0.99 (0.95-1.02)
Walkability					
Low	–	–	1.00	–	1.00
Low-medium	–	–	0.99 (0.97-1.02)	–	0.99 (0.96-1.02)
Medium-high	–	–	1.00 (0.96-1.03)	–	0.99 (0.96-1.03)
High	–	–	1.03 (0.98-1.07)	–	1.02 (0.98-1.07)
Socioeconomic disadvantage					
High	–	–	–	1.00	1.00
High-medium	–	–	–	1.00 (0.97-1.03)	0.99 (0.96-1.03)
Medium	–	–	–	1.01 (0.98-1.04)	1.01 (0.97-1.04)
Medium-low	–	–	–	1.04 (1.00-1.07)	1.03 (1.00-1.07)
Low	–	–	–	1.01 (0.97-1.05)	1.01 (0.97-1.05)
Model diagnostics					
pD	78.57	38.51	39.12	38.15	38.24
DIC	1963.23	1886.54	1889.39	1890.17	1892.30
Fit (1=best, 5=poorest)	5	1	2	3	4
Spatial fraction	0.98	0.90	0.88	0.86	0.83

PR prevalence ratio, CrI credible interval, pD effective parameters, DIC Deviance Information Criterion

Model 1 null model with expected cases proportional to the overall prevalence

Model 2 null model with expected cases adjusted for individual-level factors

Model 3 Model 2 + Sydney Walkability Index

Model 4 Model 2 + Index of Relative Socioeconomic Disadvantage

Model 5 Model 3 + Index of Relative Socioeconomic Disadvantage

Interaction models (M6) found no evidence that IRSD modified associations between SWI and prevalence of sufficient walking (*Δ*_*DIC*_=8.2), sufficient MVPA (*Δ*_*DIC*_=22.9) or high MVPA (*Δ*_*DIC*_=22.7) to improve health. However, there was strong support for the simpler sufficient walking model M3 without IRSD compared to the best fitting M5 (see Table 4). The DIC for M3 increased by only 0.7, had fewer effective parameters, and reduced spatial variation by a further 28.3% relative to M5. There was also strong support for an association between SWI and prevalence of sufficient MVPA model M3 (see Table 5). This model had a slightly increased DIC compared to the best fitting model M2 (*Δ*_*DIC*_=0.5) but fewer effective parameters and reduced postal area clustering by an additional 47.4%.

**Fig. 2:**
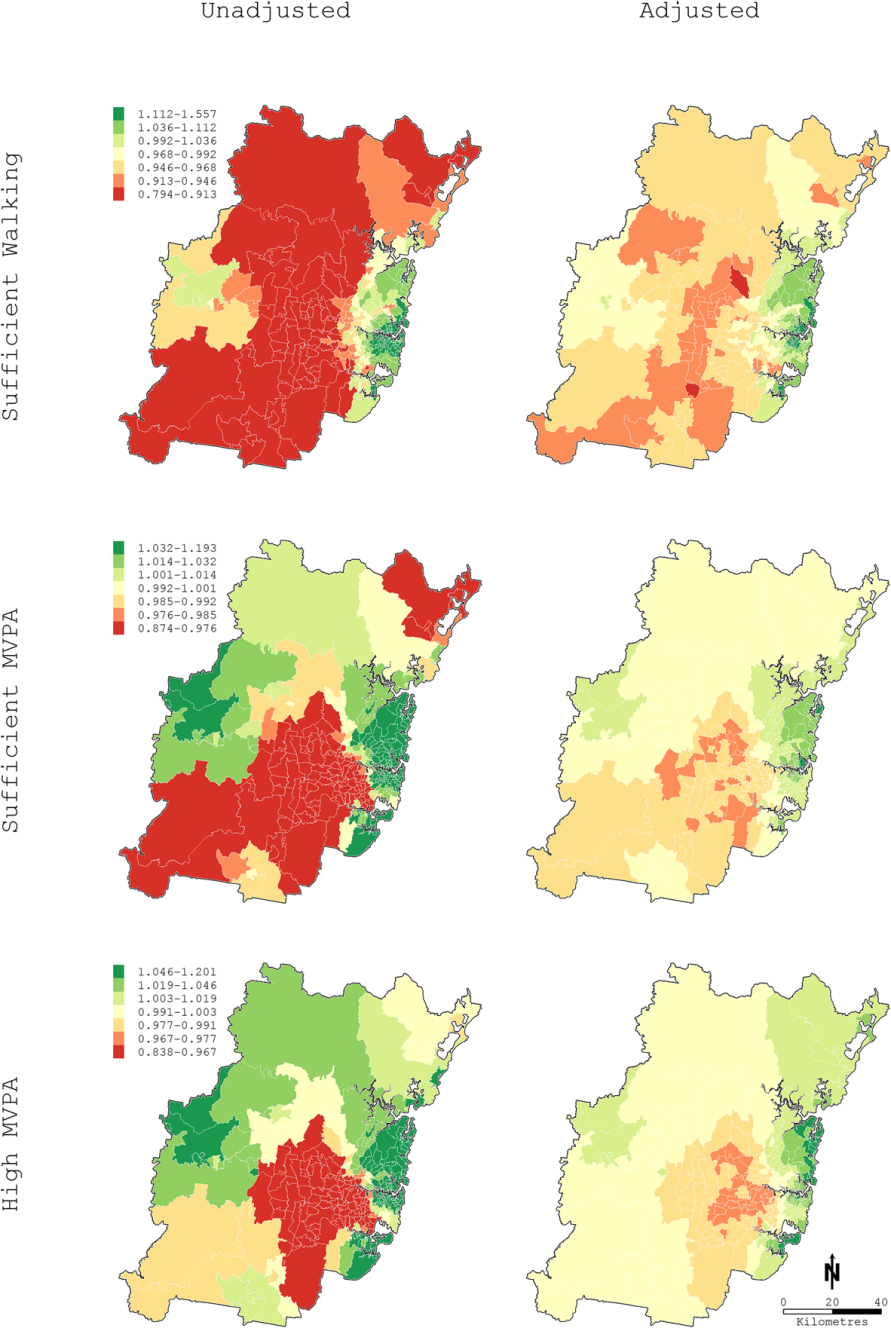
Excess prevalence ratios for physical activity outcomes in Sydney Statistical Division. Excess prevalence ratios were derived by exponentiating the sum of the log odds for the spatial and non-spatial random effects. Left panel maps report estimates from unadjusted models (Model 1) using the mean prevalence for the study area to calculate expected cases. Right panel maps report estimates from fully adjusted models including area-level walkability and relative socioeconomic disadvantage (Model 5) with expected cases derived from individual-level logistic regression models

The left-hand panels of Fig. 2 plot the residual geographic variation in unadjusted mean prevalence rate ratios (M1) for sufficient walking, sufficient MVPA, and high MVPA to improve health, which ranged from 0.79-1.56, 0.87-1.19, and 0.84-1.20, respectively. There is clear evidence for geographic clustering of postal areas with lower prevalence in southern Sydney and higher prevalence in eastern Sydney. Low rates of sufficient walking are also evident in central north Sydney, and clusters of higher rates of sufficient and high MVPA in outer western Sydney. The right-hand panels of Fig. 2 show residual prevalence rate ratios for fully-adjusted models (M5). Residual prevalence rate ratios for all outcomes were attenuated with ranges reduced to 0.90-1.25, 0.98-1.04, and 0.97-1.07 for sufficient walking, sufficient MVPA, and high MVPA, respectively. Despite these reductions, spatial fractions and disease maps indicated residual variance was principally spatial with higher prevalence on the eastern seaboard, and lower prevalence in central and southern Sydney. A north-south band of low prevalence is also evident for sufficient walking to improve health.

## Discussion

To our knowledge this is the first study to report macro-level associations between walkability and MVPA to improve health using a representative large-scale cohort and geospatial methods. Our results provide support for a positive association between increasing postal area walkability and prevalence of sufficient walking to improve health; weaker support for a positive association between increasing postal area walkability and prevalence of sufficient MVPA to improve health; and no support for an association between postal area walkability and prevalence of high MVPA. These findings are independent of individual-level demographic, social, economic, and health factors, and area-level socioeconomic disadvantage. We also found geographic clustering in prevalence of all MVPA outcomes, with higher rates of sufficient walking, sufficient MVPA, and high MVPA to improve health in the central business district and adjacent east-coast areas, and lower rates through central and southern Sydney. Approximately half of this spatial variation is explained by postal area walkability for sufficient walking and sufficient MVPA to improve health. Taken together, our findings extend individual-level environment-behavior relations between walking to improve health and walkability to population and spatial scales typically used for health planning, intervention, and surveillance; highlight the utility of spatial analysis for informing walkability research and planning; and support the validity of undertaking geographical analysis on the 45 and Up Study cohort.

### Walkability and moderate and vigorous physical activity

Syntheses of the research literature consistently report that residents in highly walkable neighborhoods are more likely to participate in MVPA, especially for active transportation, than residents in less walkable neighborhoods (see [10, 11, 84–87]). Some of these reviews have additionally concluded that the evidence is sufficiently robust to recommend incorporating built environment factors, including walkability, into urban design, transportation, and health planning [84, 85]. However, this evidence base is largely derived using individual-level studies that measure walkability at micro-environmental spatial scales [28], which has raised concerns about its validity for population-level action [32]. Our results indicate that increasing macro-environmental walkability at the postal area level is positively associated with population-levels of sufficient walking to improve health. Prevalence of sufficient walking to improve health was 12-29% greater in high versus low walkable areas, and 1-8% greater in medium-high versus low walkable areas; no difference was observed between medium-low and low walkability areas. These results indicate that micro-level associations between walkability and walking manifest at macro-level spatial scales that are similar to those used for population health planning and intervention; support the validity of individual-level walkability evidence for informing population-level action to increase walking for health; and extends to middle and older aged populations, with previous research showing increased area-level walkability is associated with higher prevalence of walking in employed populations [12, 17, 34]. Our results also provide helpful information for targeting interventions to increase walking and walkability identified in the New South Wales State Government’s plan for growing Sydney [20].

We observed a monotonically increasing exposure-response gradient between postal area walkability and prevalence of sufficient walking to benefit health. The effect size for high versus low walkability areas was three and seven times greater than for medium-high and low-medium versus low areas, respectively. This suggests a threshold effect whereby high levels of environmental walkability are required to observe an association with population-levels of sufficient walking to improve health. In our study this equated to median values of 46.0 residential dwellings per hectare, 162.5 intersections per square kilometer, and a land use entropy mix of 0.13. We have previously raised the possibility of a walkability threshold in the Sydney metropolitan region [17], and Kelly et al. have recently reported macro-level journey to work results for North American populations consistent with a threshold effect [34]. However, we are unable to preclude the possibility that any threshold is due to scale effects, which can diminish associations as spatial granularity coarsens [88]. For example, Australian research has demonstrated that associations between walkability and individual-level walking for transport attenuate as walkability is measured at increasingly coarser spatial scales [15]. However, the similarity of our results to other macro-level studies conducted at finer geographic resolutions (e.g., [17, 34]), and our matching of outcome and exposure scales, provide some evidence against a spatial scale artefact.

We found support for an association between walkability and sufficient MVPA to improve health after adjusting for individual differences. This finding was somewhat unexpected as walkability indexes are typically specific for utilitarian walking [8, 12, 17], although findings have been increasingly mixed in recent years with both positive [89] and null [15, 90] results reported. Our finding possibly reflects the very high prevalence of walking in our cohort. Sufficient MVPA to improve health was reported by 69.4% of Sydney respondents of which 45.7% attained this from walking alone. A large subset of sufficiently active walkers may also explain other positive findings for MVPA reported in the walkability literature (e.g., [89]), and reaffirms the strategy of promoting moderate-intensity walking to increase population levels of sufficient MVPA to improve health [1–4].

Our results provide no evidence for a macro-level association between postal area walkability and prevalence of high MVPA to enhance health. We defined high MVPA as ≥300 min per week, which equates to approximately 60 minutes of moderate or 30 minutes of vigorous intensity activity on most days of the week. This outcome was chosen as it is thought to reflect the minimum MVPA required for prevention of weight gain and some cancers [53]. It is likely that persons meeting this threshold would do so through a combination of moderate and high intensity physical activity, and not by walking alone. Vigorous physical activity is most consistently associated with availability of home exercise equipment and convenience of nearby facilities [91–94], which are distinctly different environmental factors to those underpinning walkability. As such, this study extends our previous findings [17] on the domain specificity of the SWI in employed populations to the general population aged 45 years and over living in Sydney, Australia.

### Geographic variation in moderate and vigorous physical activity

We observed very high levels of geographic clustering for all MVPA outcomes in unadjusted spatial analysis with increased prevalence in the Sydney central business and surrounding east-coast areas, and reduced prevalence through central and southern Sydney. Postal area walkability explained 65.8% and 47.4% of residual geographic variation in sufficient walking and sufficient MVPA to improve health but only 15.5% of high MVPA. These associations were readily apparent in disease maps, and were substantially attenuated by the inclusion of postal area walkability in spatial models. We believe presenting population-level variation in MVPA as disease maps is likely to be especially helpful for identifying and targeting areas that may benefit from infrastructure upgrades or developments, and generating hypotheses for additional research. This is supported by primary health care research showing maps are readily comprehended by decision-makers, and can facilitate the alignment of services, and interventions with population needs [95].

Ours appears to be the first built environment study to quantitatively demonstrate associations between area-level walkability and spatial patterning in population-levels of sufficient walking and MVPA to improve health. Merom and colleagues have previously reported geographic variation in prevalence and increases in prevalence in any walking for Sydney local government areas between 2002–2012 [28]. They also observed highest prevalence in the Sydney business district and adjacent areas, and lowest prevalence through central and southern Sydney. Our findings expand on this research in a number of aspects. First, our study used fully Bayesian hierarchical models to account for, and leverage, spatial autocorrelation to produce “smoothed” effect estimates, valid credible intervals, and partition variation into spatially structured and unstructured components. Second, we used MVPA outcomes that are routinely used to monitor health status in Australian populations. Third, we conducted our study at a much finer spatial resolution in order to maximize between-area heterogeneity and increase the locational specificity of our results [96]. Fourth, we evaluated the proportion of excess spatial variation in MVPA outcomes that was attributable to postal area walkability after removing variation due to individual and area level demographic, social, economic, and health status factors. Together, these differences allowed us to produce robust disease maps over a shorter time horizon and identify regions where walkability may be contributing to population differentials in sufficient MVPA to improve health.

We also observed spatial clustering in high MVPA to improve health but this was unrelated to postal area walkability. Variation in high MVPA to enhance health is most consistently associated with availability of home exercise equipment and convenience of nearby facilities [91–94]. There is also some evidence that higher densities of exercise facilities within 1,000 metres of an individual’s residence is associated with increased duration of MVPA and odds of meeting physical activity recommendations [97]. The extent to which the observed geographic variation in high MVPA in Sydney is attributable to the spatial distribution of these and other environmental factors was beyond the scope of our study but could easily be addressed by including density estimates into our analytic framework, and warrants further investigation given the hypothesised benefits of high MVPA for the prevention of weight gain and some cancers [53].

Excess prevalence for all outcomes decreased after adjusting for individual-level factors; however, despite these reductions disease maps remained highly clustered, especially for walking. This finding has important methodological implications because it suggests (1) spatial autocorrelation is an inherent feature of built environment data, and (2) individual differences do not fully explain this clustering. Spatial autocorrelation is a problem for linear regression because it violates the assumption that residuals are independent and identically distributed, which may lead to erroneous inference [98]. Concerns regarding ‘spatial multicollinearity’ in the walkability literature are not new but have focused on the covariation between built environment factors [99]. For example, NQLS-based walkability indexes aggregate data across environmental variables to minimize multicollinearity and leverage their ‘synergy’ [12]. However, this does not account for spatial autocorrelation in the distributions of outcome or study factors, which may be substantial based on our results.

Multilevel analysis using general and generalized linear mixed models (GLMM) provides one solution to account for spatial autocorrelation, and is already used widely in the built environment literature for individual-level analyses where walkability is measured at meso-levels [10]. There are two problems with this approach: first, researchers typically use GLMM only when walkability is measured at a level different to that used for inference, and are unlikely to consider more complex analytical models for apparently non-hierarchical designs; and second, GLMM most often employ a covariance structure that conflates spatial and non-spatial variation within a single variance component [100]. Our study highlights the utility of examining this spatial component for informing policy and planning activities. We therefore recommend the use of spatial models in built environment research to (1) make explicit the expectation of spatial structure in the environment-behavior data under investigation; and (2) identify geographic variation in outcomes to inform population-level programming. We believe the Bayesian disease mapping and ecological regression approach used in our study is especially useful in this regard because it incorporates both individual and area-level factors; is easily implemented in freely available statistical software; and will provide unbiased effect estimates in the absence of spatial variation [101].

### Area-level socioeconomic disadvantage and Moderate and Vigorous Physical Activity

Area-level relative socioeconomic disadvantage was not identified as a correlate, confounder or effect modifier in any of our walking or MVPA models. This differs from our previous study that found median household income was independently associated with walking to work, and attenuated associations between SWI and walking to work at the smaller Census Collection District level in Sydney metropolitan region [17]. Kelly and colleagues have reported similar findings for block group prevalence of walking to work in the St. Louis City and County areas of North America [34]. These differences likely reflect methodological improvements in our current study, including adjustment for individual-level factors to account for heterogeneity in the demographic, social, economic, and health characteristics of postal area respondents. Our findings therefore extend empirical observations that area-level socioeconomic status is unrelated to individual-level walking and walkability after adjusting for person-level factors [90] to population-level associations between MVPA and walkability, and reaffirm the potential of walking to address population inequalities in MVPA participation [102].

### Implications for policy and planning in Sydney

The Sydney environment will be transformed over the next 20 years through the *Plan for Growing Sydney* [20], which aims to accommodate a projected population increase of one million people by developing communities that are strong, healthy, and well connected. Creating local opportunities for transport-related walking through strategic land use and infrastructure developments is a key strategy of this plan [20]. Ensuring these developments and upgrades maximize transport, health, and environmental benefits will be a significant challenge for population health advocates. Our study indicates that participation in sufficient walking to improve health is not uniform across Sydney but varies geographically. This structure is independent of individual-level demographic, social, environmental, and economic factors, and unrelated to area-level socioeconomic disadvantage—factors often considered instrumental in urban design, transportation, and health policy and planning (e.g., [20, 103]). The SWI has the potential to inform these decisions by characterizing the walkability of geographic areas with lower than expected MVPA participation rates with a view to prioritizing infrastructure upgrades and developments that support active transportation and walking for other purposes [8, 20].

### Strengths and limitations

This study has a number of strengths. First, we linked the SWI to high-quality and geocoded baseline data from the 45 and Up Study, which allowed us to examine area-level associations between walking infrastructure and population-levels of MVPA adjusting for individual-level factors. The 45 and Up Study is a prospective cohort with approximately quinquennial follow-up [47]. As these follow-up data are collected, geocoded, and made available to researchers, they will provide unique opportunities to examine the influence of walkability on the walking behavior and health outcomes of individuals and populations with increasingly sophisticated designs [17]. Second, we measured walkability using the SWI [17], which is derived from NQLS [12] and PLACE [8] walkability indexes. These indexes form the basis of an extensive national and international literature linking increased environmental walkability to individual-levels of sufficient moderate-intensity physical activity to enhance health [17], and provides an international context for our findings and methodological approach for population-level programming and built environment research. Third, we used a Bayesian disease mapping and ecological regression study design that allowed us to quantify the geographic variation in MVPA outcomes attributable to postal area walkability after removing variation explained by other individual and area-level factors. Our approach appears novel in the walkability literature despite its common use in epidemiology for small area estimation problems [104, 105]. A particular advantage of this approach is the capacity to produce smoothed disease maps to communicate variation in geographic risk to politicians, planners, and policymakers. Fourth, we used a spatial scale more proximal to those typically used for population health planning and status monitoring [28] but within the upper range of buffers used for individual-level analyses [56, 57]. However, we caution against interpreting area-level walkability as simply an average of individual-level exposures within the areal unit. Area-level walkability is derived at the spatial scale of analysis. It is a contextual measure of the area’s built environment to which groups, communities and populations are exposed. That is, we maintain area-level walkability is a characteristic of the areal unit and measures an aspect of walkability that is qualitatively different to individual-level walkability. Finally, we observed that unweighted prevalence ratios used in our spatial analyses were analogous to those derived using post-stratification weighting, which provides support for the generalizability of our findings and spatial trends to the Sydney Statistical Division.

Our study is also subject to a number of limitations. First, our study sample included all Sydney respondents to the 45 and Up Study with complete data on selected baseline survey items. Although the 45 and Up Study includes approximately 10% of the NSW Population aged ≥45 years, the cohort is younger, more highly educated, and more likely to speak English at home and live with a partner than the Sydney study base [43]. Our sub-sample reflected these cohort characteristics. Point estimates may be biased by non-response in cohort studies [106, 107]; however, relative measures of effect are generally considered representative of the study base [108–112]. The external validity of 45 and Up Study results is supported by research showing that relative effect measures derived from this cohort are consistent with those from population-representative surveys [79], irrespective of sample weighting. The very high correlations between unweighted and weighted postal area prevalence estimates described in our study are consistent with these observations, and provide support for the external validity of our findings.

Second, we used a two-step approach to model associations between postal area physical activity and walkability using ecological spatial regressions that were adjusted for person-level factors by including model offsets derived from individual-level regressions. This approach is often used for spatial analyses where area and individual-level factors cannot be incorporated into a single parsimonious model (see [66–68, 113]); however, both individual and area-levels factors would ideally be modeled concurrently. These types of multilevel spatial models are starting to appear in the methodological literature (see [114, 115]) but are not yet easily implemented in standard statistical packages and require distributed computational environments for large data problems [115], which prohibited their use in our study.

Third, although widely used for environment-behavior research, NQLS-based indexes are derived using population-specific cut-points for defining walkability quartiles, which can result in data-dependent exposure categories [116–118]. To increase the utility of our findings for planners, policy analysts, and researchers, we have reported the median, minimum, and maximum environmental variable scores associated with walkability quartiles in our study area. These values compare favorably with the limited number of studies that also report environmental values within walkability quartiles (see [21, 54, 119]), and provides support for the international relevance of our findings. A small number of recent studies have directly modeled associations between NQLS environmental variables and minutes spent in moderate-intensity physical activity (e.g., [18, 120, 121]). These studies employed non-parametric generalized additive models (GAM) with spline functions to account for the complex, non-linear relationships between exposure and outcome variables. This was beyond the scope and resources of our study, which would have required fitting computationally intensive penalized spline terms to our already resource-demanding conditional autoregressive models. We therefore recommend that analysts and researchers carefully consider the concordance of built environment characteristics between their target environments and the Sydney metropolitan area before utilising our findings for population-level policy, planning, and intervention.

Fourth, we were unable to evaluate if our results were sensitive to the spatial scale at which area-level walkability is measured because our access to geocoded data was limited to postal area identifiers. The modifiable areal unit problem has the potential to affect all spatial analyses that do not have access to individual-level longitude and latitude coordinates [122]. However, the concordance of our results with previous macro-level studies conducted at finer spatial resolutions (see [12, 17, 34]) supports the robustness of our findings. Fifth, postal areas are Australian Census statistical output units, so their geographic dimensions and populace may not be representative of planning geographies used in other countries. Finally, we used a cross-sectional design which precluded considerations of causality.

## Conclusion

Our study appears to be unique in the walkability literature for its population focus and spatial approach. We observed that increasing postal area walkability was associated with higher prevalence of sufficient walking and sufficient MVPA to benefit health in Sydney, and accounts for large proportions of the residual geographic variation in these outcomes that remains after adjusting for individual and area-level demographic, social, economic, and health factors. Our results support the potential of walkability indexes to inform and target population-level programming, especially if local context is incorporated into these activities, and affirms the importance of including “place” in walkability research and planning to ensure the robustness of outcomes. From a practical perspective, our study demonstrates the utility of disease maps for communicating adverse geographic risk of MVPA outcomes and the extent to which this may be attributable to modifiable environmental factors such as walkability. Finally, our findings provide another resource for the NSW Government to use in sustainably growing Sydney by identifying regional opportunities for strategic land use and infrastructure developments to increase population-levels of walking and create built environments that support health.

## Abbreviations

CI: Confidence interval
CrI: Credible interval
DIC: Deviance information criterion
GLMM: Generalized linear mixed models
IRSD: Index of relative socioeconomic disadvantage
M1–M6: Models 1–6
MVPA: Moderate and vigorous physical activity
NQLS: Neighborhood quality of life study
NSW: New South Wales
pD: Effective parameters
PR: Prevalence ratio
SEEF: Social, environmental and economic factors
SWI: Sydney Walkability Index
